# Influence of Tissue Type on the Bacterial Diversity and Community in Pork Bacon

**DOI:** 10.3389/fmicb.2021.799332

**Published:** 2021-12-03

**Authors:** Wenjuan Gong, Yan Zhu, XiXiong Shi, Weibing Zhang, PengCheng Wen

**Affiliations:** College of Food Science and Engineering, Gansu Agricultural University, Lanzhou, China

**Keywords:** bacon, bacterial community, diversity, high-throughput sequencing, pork

## Abstract

In current study, bacterial diversity and community in different tissues of pork bacon were determined using high-throughput sequencing. In total, six phyla and 111 bacterial genera were identified. Among them, three dominant genera (*Staphylococcus*, *Acinetobacter*, and *Macrococcus*) were shared by all bacon samples. The linear discriminant analysis showed that 24 bacterial taxa significantly differentiated between the tissues. Results of non-metric Multidimensional Scaling and redundancy analysis showed that physicochemical characteristics of the tissue prominently structured the bacterial communities. Network analysis also illustrated that tissue type was an important factor impacting the bacterial interactions in different types of tissue. The results of current study can add valuable insights to the traditional homemade pork bacon.

## Introduction

Different types of traditional meat products with special flavor, including sausage, fermented fish, and fermented pork fat, have been prepared and consumed for hundreds of years (Xiao et al., 2013; Yi et al., 2016; De Mandal et al., 2018; Zang et al., 2018). Among them, Chinese pork bacon has a very long history in many provinces in China (Guo et al., 2016; Wang et al., 2019). A variety of Chinese traditional pork bacon styles exist in China including Sichuan, Hunan, Guangdong, Jiangxi, and Yunnan styles (Wang et al., 2019). This product is rich in protein and fat, and also contains phosphorus, potassium, sodium, and other elements, and is used in many traditional dishes. Due to its unique flavor, delicious taste, and particular texture, it is made and favored by the locals (Yu and Sun, 2005).

In the traditional process of pork bacon, raw meat is exposed to the air, so abundant and diverse microorganisms may colonize it and grow (Doulgeraki et al., 2012). Various lactic acid bacteria, *Staphylococcus*, and *Macrococcus*, have been found on the surface of bacon (Yi et al., 2016). They can secrete various enzymes such as proteases, lipases, and nitrate reductase, which will degrade proteins, lipids, and other composition in the meat into ketones, esters, and acids, and contribute to the unique aroma and taste characteristics of the end products (Li et al., 2013; Yi et al., 2016). Some species such as *Staphylococcus xylosus* can endow the products with red color and texture (Li et al., 2013; Hu et al., 2018). Therefore, it’s necessary to illustrate the bacterial community of traditional meat products.

Since fresh streaky pork used for bacon is not divided, the bacon includes three parts: fat, lean meat and skin. In the previous study, we found obvious differences in physicochemical properties of the different tissues, which led to significant differences in the distribution of fungi (Zhang et al., 2021). However, there have been few reports on the bacterial community in different tissues of bacon. In the current study, our objective was to investigate the diversity of bacterial community in different types of bacon tissue.

## Materials and Methods

### Sample Collection

**FIGURE 1:**
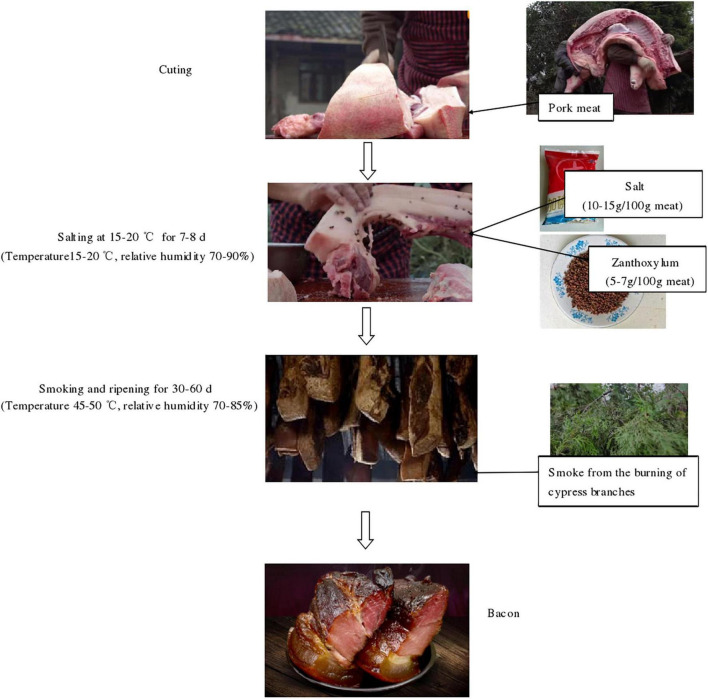
A schematic diagram of the production of homemade pork bacon.

Bacon samples were collected directly from six local producers in Lacquer Tree village, Fan Kuai town, Xuanhan County and Dazhou City (Sichuan Province, China). The processing technology of the bacon is presented in Figure 1. All the samples were collected according to the method previously described by Zhang et al. (2021). The samples were packed in sterile bags and transported to the laboratory. In sampling, bacon tissues around 2 mm from the surface were sliced using a sterilized knife. Based on the tissue type, the samples are divided into three groups: F group (the adipose tissue), M group (the muscle tissue), P group (pork skin). Then, the newly collected bacon samples were used for microbial enumeration and the samples used for DNA extraction were stored at -80°C.

### Microbial Enumeration and Identification

According to a previous method described by Zhang et al. (2021), microbial enumeration was conducted by gradient dilution method on selective medium: Total viable counts (TVC) on Plate Count Agar (PCA, Oxoid) incubated at 30°C for 72 h; Staphylococcaceae on Baird-Parker Agar (BP, Oxoid), incubated at 37°C for 48 h; Enterobacteriaceae on Violet Red Bile Glucose Agar (VRBGA, Lang Bridge), incubated at 37°C for 36 h; *Pseudomonads* on Centrimide-Fucidin-Cepha Loridine medium (CFC, Oxoid), incubated at 25°C for 48 h; *Brochothrix* on Streptomycin sulfate thallous acetate cycloheximide (actidione) agar medium (STAA, Oxoid), incubated at 25°C for 48 h.

About 10 presumptive isolates were selected according to the morphological features on the plates. Then all the strains were identified based on 16S rRNA analysis. The bacterial genomic DNA of the strains was extracted by TIANamp DNA Kit (DP302) (Tiangen, Beijing, China). The 16S rRNA gene fragment of the selected bacteria was amplified through the universal primers 27F (5′- AGAGTTTGATCCTGGCTCAG-3′) and 1492R (5′-AAGGAGGTGATCCAGCCGCA-3′). PCR amplification programs were conducted according to the previous method (Li et al., 2019). The PCR products were checked using 0.8% (w/v) agarose gelelectrophoresis. The gel was visualized using an Image Master^®^ VDS. DNA sequence analysis was performed by GenScript (Nanjing, Jiangsu, China). The sequence identity was analyzed using the Blastn program against the GenBank database, and sequences with an identity threshold of 97% were downloaded for further analysis.

### DNA Extraction, PCR Amplification, and Sequencing

Microbial genomic DNA was extracted from the tissues referring to the previous method detailed in Zhang et al. (2021). The V3–V4 region of 16S rRNA was amplified using universal primers (338F and 806R). The library was constructed and the isolated DNA was sequenced at Biomarker Technologies Corporation in Beijing. The raw sequence data were deposited to NCBI, and the accession number was SAMN14260270-14336709.

### Data Preprocessing and Analysis

Quality control and analysis of the raw reads were mainly performed by QIIME (V1.7.0). Subsequently, Mothur (V.1.31.2) was applied to remove chimeras. Operational taxonomic units (OTUs) were recovered from quality sequences with similarity larger than 97% using the UCLUST method in QIIME. Subsequently, OTUs taxonomy was annotated by a QIIME-based wrapper of RDP-classifier (v.2.2) against the RDP bacterial 16S rRNA database (Release 11.1). Three coefficients of alpha diversity were calculated for each sample using the Mothur software (V.1.31.2). Duncan’s test was used to identify the significant differences between the different tissues in alpha indexes. Non-metric Multidimensional Scaling (NMDS) were constructed using the R package (v2.15.3^1^). The Adonis permutational multivariate analysis (Adonis/PerMANOVA) and analysis of similarities (ANOSIM) were conducted according to the method previously described by Lozupone et al. (2007). The cluster analysis was conducted with unweighted pair-group method using arithmetical averages (UPGMA). LEfSe was conducted using the online Galaxy work flow framework (LDA score ≥ 4.0 and *p* ≤ 0.05)^2^ (Segata et al., 2011). The SparCC algorithm was applied to investigate the correlation of all bacterial genera. Correlation networks and redundancy analysis (RDA) were made using the Biomarker biocloud tools (Luo et al., 2020; Zhang et al., 2021). Data of the physicochemical characterization used for RDA was showed in our previous study (Zhang et al., 2021).

## Results

### Microbial Enumeration and Identification of the Isolates

As shown in Table 1, total viable count on PCA was lowest in the M group. No significant difference (*P* > 0.05) was found for colonies on BP between the three groups. Colonies of the M group on VRBGA were significantly lower than those of others (*P* < 0.05). Colonies of the F group on CFC were higher than that of the P group and no visible colonies were observed in samples of the M group. Colonies of the F group on STAA were lower than that of the P group and no visible colonies were observed in samples of the M group.

**TABLE 1:** Viable counts of bacteria in the samples.

Sample ID	Total viable count log_10_ CFU/g	Staphylococcaceae log_10_ CFU/g	Enterobacteriaceae log_10_ CFU/g	Pseudomonas log_10_ CFU/g	Brochothrix log_10_ CFU/g
M1	5.31 ± 0.09^a^	5.05 ± 0.12^a^	1.21 ± 0.04^a^	ND	ND
M2	5.26 ± 0.05^a^	4.85 ± 0.09^ab^	1.09 ± 0.03^ab^	ND	ND
M3	5.29 ± 0.08^a^	5.15 ± 0.08^a^	1.18 ± 0.05^a^	ND	ND
M4	5.23 ± 0.19^a^	4.95 ± 0.06^a^	1.13 ± 0.04^a^	ND	ND
M5	5.27 ± 0.18^a^	5.21 ± 0.05^a^	1.09 ± 0.05^ab^	ND	ND
M6	5.25 ± 0.16^a^	5.17 ± 0.11^a^	1.07 ± 0.03^ab^	ND	ND
M_*mean*_	5.27 ± 0.09^b^	5.06 ± 0.11^a^	1.13 ± 0.06^b^	ND	ND
F1	6.63 ± 0.18^a^	4.85 ± 0.12^ab^	3.35 ± 0.08^a^	4.73 ± 0.09^a^	1.15 ± 0.08^a^
F2	6.57 ± 0.31^a^	4.96 ± 0.06^a^	3.49 ± 0.05^a^	4.89 ± 0.15^a^	1.09 ± 0.13^a^
F3	6.49 ± 0.28^a^	4.91 ± 0.13^a^	3.56 ± 0.08^a^	4.81 ± 0.23^a^	1.12 ± 0.09^a^
F4	6.56 ± 0.34^a^	4.89 ± 0.25^ab^	3.59 ± 0.12^a^	4.79 ± 0.18^a^	1.17 ± 0.05^a^
F5	6.68 ± 0.05^a^	5.02 ± 0.19^a^	3.41 ± 0.09^a^	4.83 ± 0.09^a^	1.18 ± 0.15^a^
F6	6.55 ± 0.23^a^	5.08 ± 0.09^a^	3.38 ± 0.06^a^	4.86 ± 0.11^a^	1.21 ± 0.31^a^
F_*mean*_	6.58 ± 0.07^a^	4.95 ± 0.09^a^	3.46 ± 0.10^a^	4.82 ± 0.06^a^	1.15 ± 0.04^b^
S1	6.78 ± 0.19^a^	4.97 ± 0.09^a^	3.59 ± 0.08^a^	1.31 ± 0.05^a^	4.25 ± 0.05^a^
S2	6.83 ± 0.09^a^	5.11 ± 0.05^a^	3.46 ± 0.15^a^	1.28 ± 0.09^a^	4.39 ± 0.16^a^
S3	6.65 ± 0.29^a^	4.98 ± 0.08^a^	3.41 ± 0.19^a^	1.25 ± 0.07^a^	4.32 ± 0.05^a^
S4	6.91 ± 0.09^a^	5.09 ± 0.05^a^	3.53 ± 0.07^a^	1.21 ± 0.06^a^	4.29 ± 0.08^a^
S5	6.69 ± 0.11^a^	5.02 ± 0.19^a^	3.56 ± 0.13^a^	1.19 ± 0.09^a^	4.31 ± 0.09^a^
S6	6.76 ± 0.09^a^	4.85 ± 0.07^ab^	3.58 ± 0.06^a^	1.25 ± 0.07^a^	4.45 ± 0.21^a^
S_*mean*_	6.77 ± 0.09^a^	5.00 ± 0.09^a^	3.52 ± 0.12^a^	1.25 ± 0.08^b^	4.34 ± 0.08^a^

*M1–M6, sample from the muscle tissue of bacon; F1–F6, sample from the adipose tissue of bacon; S1–S6, sample from pork skin of bacon. Lowercase letters indicate Duncan’s pairwise differences between samples from different producers (p < 0.05). ND, Not detected.*

Overall, 64 colonies were obtained from the selective culture media, the numbers were 28 (PCA), 13 (BP), 7 (VRBGA), 11 (CFC), and 5 (STAA), respectively. Then all the strains were identified by 16S rRNA and sequences similarities of isolates with representative strains were shown in Table 2. 12 isolates of 28 (42.8%) were identified as *Staphylococcus xylosus* and *Staphylococcus vitulinus* on PCA medium. Other isolated strains were clustered into five species and the most adequate species were *Macrococcus caseolyticus* and *Acinetobacter baumannii*, followed by *Pseudomonas* sp., *Brochothrix* sp., and *Carnobacterium divergens*. 92.3% isolates were identified as *Staphylococcus xylosus* (6 isolates) and *Staphylococcus vitulinus* (6 isolates) from BP medium. Another isolated microorganism from BP was *Carnobacterium divergens* (1 isolate), which was detected only in pork skin. Seven isolates from VRBGA medium were identified as *Psychrobacter* sp. (3 isolates), *Carnobacterium maltaromaticum* (2 isolates), and *Carnobacterium divergens* (2 isolates), which were found only in pork skin. *Macrococcus caseolyticus* (4 isolates) and *Pseudomonas* sp. (7 isolates) were found on CFC plates. *Brochothrix campestris* (2 isolates) and *Brochothrix* sp. (3 isolates) was the isolated microorganisms from STAA, which were detected only in pork skin.

**TABLE 2:** Identification of isolates based on 16S rRNA gene sequencing analysis.

Medium	Isolates	Base pairs	Closest relative	Accession no.	Identity (%)	Bacon tissue		
						F	M	S
PCA	P1	1456	*Staphylococcus xylosus*	NR_036907	99	2	3	1
	P2	1272	*Staphylococcus vitulinus*	MT760110	99	2	3	1
	P3	782	*Macrococcus caseolyticus*	KX246813	99	1	1	3
	P4	913	*Acinetobacter baumannii*	MT138560	100	3	1	1
	P5	1351	*Pseudomonas* sp.	KX186983	98	2	0	0
	P6	1315	*Brochothrix* sp.	HQ890945	99	0	0	2
	P7	1367	*Carnobacterium divergens*	MN229536	99	0	0	2
BP	P1	1456	*Staphylococcus xylosus*	NR_036907	99	2	3	1
	P2	1272	*Staphylococcus vitulinus*	MT760110	99	2	3	1
	P7	1367	*Carnobacterium divergens*	MN229536	99	0	0	1
VRBGA	V1	1098	*Psychrobacter* sp.	FR717284	99	0	0	3
	V2	1295	*Carnobacterium maltaromaticum*	MN179346	99	0	0	2
	P7	1367	*Carnobacterium divergens*	MN229536	99	0	0	2
CFC	P5	1351	*Pseudomonas* sp.	KX186983	98	3	0	1
	P3	782	*Macrococcus caseolyticus*	KX246813	99	3	1	3
STAA	S1	1382	*Brochothrix campestris*	NR_044824	98	0	0	2
	P6	1315	*Brochothrix* sp.	HQ890945	99	0	0	3

*M, sample from the muscle tissue of bacon; F, sample from the adipose tissue of bacon; S, sample from pork skin of bacon.*

### Sequencing and Classification

568,540 high quality sequencing reads were generated across the tissues, with an average of 31,585 reads per sample (Table 3). Overall, 16,287 OTUs were generated, with an average of 904 OTUs per sample. The OTUs were significantly lower in the M group (483 OTUs) than in the other two groups (1,085 and 1,145 OTUs for F and S groups) (*P* < 0.05). However, OTUs did not differentiate between F and S groups (*P* > 0.05).

**TABLE 3:** Reads, OTUs, Good’s Coverage, Chao1, and Shannon’s indices for 16S r RNA sequencing of the bacon samples.

Group	Reads		OTUs		Good’s coverage		Chao1		Shannon	
	Mean	*SD*	Mean	*SD*	Mean	*SD*	Mean	*SD*	Mean	*SD*
M	31,778	3,408	483	73^b^	99.25%	0.11%	552.2	73.4^b^	4.77	0.29^c^
F	32,472	3,690	1,085	176^ab^	99.61%	0.08%	1149.1	38.8^a^	6.91	0.44^b^
S	30,505	1,903	1,145	173^a^	99.88%	0.06%	1146.6	73.9^a^	7.51	0.37^a^

*M, sample from the muscle tissue of bacon; F, sample from the adipose tissue of bacon; S, sample from pork skin of bacon. Lowercase letters indicate Duncan’s pairwise differences between samples from different tissue of bacon (p < 0.05).*

### Overall Characteristics of Bacterial Community

There were six bacterial phyla present in the samples, three of which were dominant in the tissues (relative abundance > 1%). Firmicutes was the most abundant bacterial phylum in all samples, with a relative abundance range of 57.68–96.44% (Table 4). Proteobacteria was the second most dominant bacterial phylum in all groups, accounting for 3.37% of the sequences in the M group, but for 29.60 and 36.29% in F and S groups, respectively. Actinobacteria was the third most predominant phylum detected in F and S groups, with a relative abundance of 2.02–4.55%. The relative abundances of the other three bacterial phyla (Bacteroidetes, Chloroflexi, and Deinococcus-Thermus) were less than 1%.

**TABLE 4:** Distributions of predominant bacteria at different taxonomic levels.

Predominant bacteria	Percentage composition in samples		
	F n = 6	M n = 6	S n = 6
**Phylum**			
Firmicutes	67.89%	96.44%	57.68%
Proteobacteria	29.60%	3.37%	36.29%
Actinobacteria	2.02%	0.14%	4.55%
**Class**			
Bacilli	67.27%	96.37%	55.82%
Gammaproteobacteria	28.72%	3.28%	34.54%
Actinobacteria	2.02%	0.14%	4.50%
Alphaproteobacteria	0.88%	0.08%	1.72%
Clostridia	0.63%	0.07%	1.79%
**Order**			
Bacillales	65.93%	96.15%	51.96%
Pseudomonadales	23.23%	2.67%	25.24%
Betaproteobacteriales	3.61%	0.45%	7.16%
Micrococcales	1.67%	0.11%	3.62%
Lactobacillales	1.33%	0.22%	3.85%
Enterobacteriales	1.56%	0.13%	1.64%
Clostridiales	0.63%	0.07%	1.79%
**Family**			
Staphylococcaceae	64.96%	96.04%	43.49%
Moraxellaceae	9.34%	2.47%	21.88%
Pseudomonadaceae	13.89%	0.20%	3.35%
Burkholderiaceae	3.61%	0.45%	7.16%
Listeriaceae	0.82%	0.08%	8.25%
Micrococcaceae	1.28%	0.08%	2.75%
Enterobacteriaceae	1.56%	0.13%	1.64%
Streptococcaceae	0.73%	0.08%	1.16%
Carnobacteriaceae	0.22%	0.02%	1.51%
**Genus**			
*Staphylococcus*	60.74%	92.38%	35.99%
*Psychrobacter*	1.36%	0.13%	16.46%
*Pseudomonas*	13.89%	0.20%	3.35%
*Acinetobacter*	7.85%	2.32%	5.34%
*Macrococcus*	4.21%	3.66%	7.48%
*Brochothrix*	0.82%	0.08%	8.25%
*Cupriavidus*	2.31%	0.22%	3.26%
*Massilia*	1.16%	0.21%	3.59%
*Carnobacterium*	0.19%	0.02%	1.44%

*M, sample from the muscle tissue of bacon; F, sample from the adipose tissue of bacon; S, sample from pork skin of bacon.*

There were 111 genera found in the bacon samples (Table 4). Overall, nine genera were dominant in the tissues, with relative abundances > 1%. Among these, three dominant genera (*Acinetobacter*, *Macrococcus*, and *Staphylococcus*) were shared by all bacon samples. In addition to the three dominant genera shared by all samples, four dominant genera (*Cupriavidus*, *Massilia*, *Pseudomonas*, and *Psychrobacter*) were both present in P and F groups. In samples of the M group, three genera (*Staphylococcus*, *Macrococcus*, and *Acinetobacter*) were dominant, with relative abundances of 92.38, 3.66, and 2.32%, respectively. Seven dominant genera (*Staphylococcus*, *Pseudomonas*, *Acinetobacter*, *Macrococcus*, *Cupriavidus*, *Psychrobacter*, and *Massilia*) occurred in the F group, with relative abundances of 60.74, 13.89, 7.85, 4.21, 2.31, 1.36, and 1.16%, respectively; *Acinetobacter*, *Pseudomonas*, and *Staphylococcus* represented 82.49% of the bacterial population. Nine dominant genera were present in the S group: *Staphylococcus* was the most dominant bacterial genus, with a relative abundance of 35.99%, followed by *Psychrobacter* (16.46%), *Brochothrix* (8.25%), *Macrococcus* (7.48%), *Acinetobacter* (5.34%), *Massilia* (3.59%), *Pseudomonas* (3.35%), *Cupriavidus* (3.26%), and *Carnobacterium* (1.44%). In additional, 102 non-dominant genera were detected in the tissues.

### Comparison of Bacterial Diversity and Communities

To assess alpha diversity, Shannon and Chao1 indexes were calculated and showed in Table 3. The lower and higher Shannon indexes were 4.77 and 7.51 for the M and S groups, respectively, indicating that bacterial diversity was lowest in the M group. The smallest Chao1 index was 552.2 for the M group on average. The Chao1 indexes did not significantly differ between P (1,146.6) and F (1,149.1) groups (*P* > 0.05). Results of the Chao1 index were consistent with the differences in OTUs of the groups (Table 3).

**FIGURE 2:**
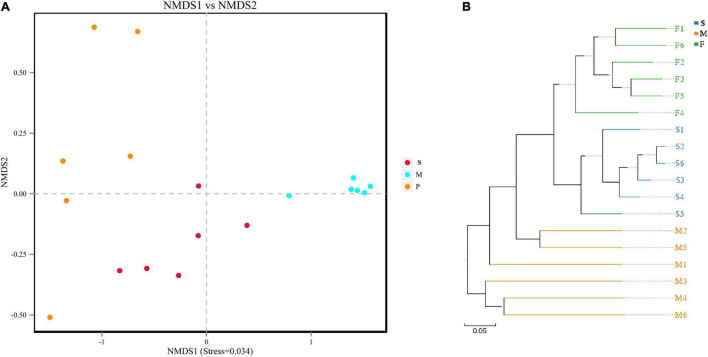
The beta diversity results of NMDS indicating the data distribution of bacon samples **(A)**; the unweighted pair-group analysis (UPGMA) based on UniFrac distance for bacterial communities of bacon samples **(B)**.

Results of NMDS illustrated the data distribution of bacon samples (Figure 2A). Samples from different tissues are separated indicating that the bacterial community of F, M, and S groups had a high degree of similarity. The results were very similar to that of unweighted pair-group analysis (Figure 2B). In addition, samples in S group are more tightly clustered than those in the F and M groups (Figure 2A). The Adonis method (PerMANOVA) was used for comparison between groups showed that *P* = 0.001 (*P* < 0.05). Results of ANOSIM illustrated that the tissue type was an important factor impacting the bacterial composition of the different tissues (*R* = 0.891, *P* = 0.002).

**FIGURE 3:**
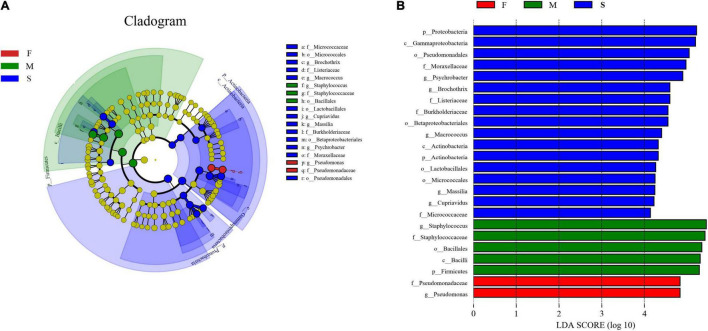
Cladogram indicating the phylogenetic distribution of taxon associated with the three groups of bacon samples. Differences are represented in the color of the most abundant class. The diameter of each circle is proportional to the abundance of the taxon **(A)**; histogram of differentially abundant features between groups (logarithmic LDA score ≥ 4.0 and *p* ≤ 0.05) **(B)**.

24 significantly different taxa between the groups were showed in Figure 3. In the M group, five taxa (Firmicutes, Bacilli, Bacillales, Staphylococcaceae, and *Staphylococcus*) were enriched and had LDA values above 5.0 (Figure 3B). In the F group, the bacterial taxa enriched were one family (Pseudomonadaceae) and one genus (*Pseudomonas*), both with LDA values higher than 4.8 (Figure 3B).

In the S group, 17 significantly different taxa were obtained. Among them, one class (Actinobacteria), one order (Micrococcales), and one family (Micrococcaceae) belonged to phylum Actinobacteria. One order (Lactobacillales), one family (Listeriaceae), and two genera (*Brochothrix* and *Macrococcus*) belonged to phylum Firmicutes. One class (Gammaproteobacteria), two orders (Betaproteobacteriales and Pseudomonadales), two families (Burkholderiaceae and Moraxellaceae), and three genera (*Cupriavidus*, *Psychrobacter*, and *Massilia*) belonged to phylum Proteobacteria. Among them, Proteobacteria, Gammaproteobacteria, and Pseudomonadales had LDA values above 5.0 (Figure 3B).

### Interactions of Bacteria in Bacon Samples

**FIGURE 4:**
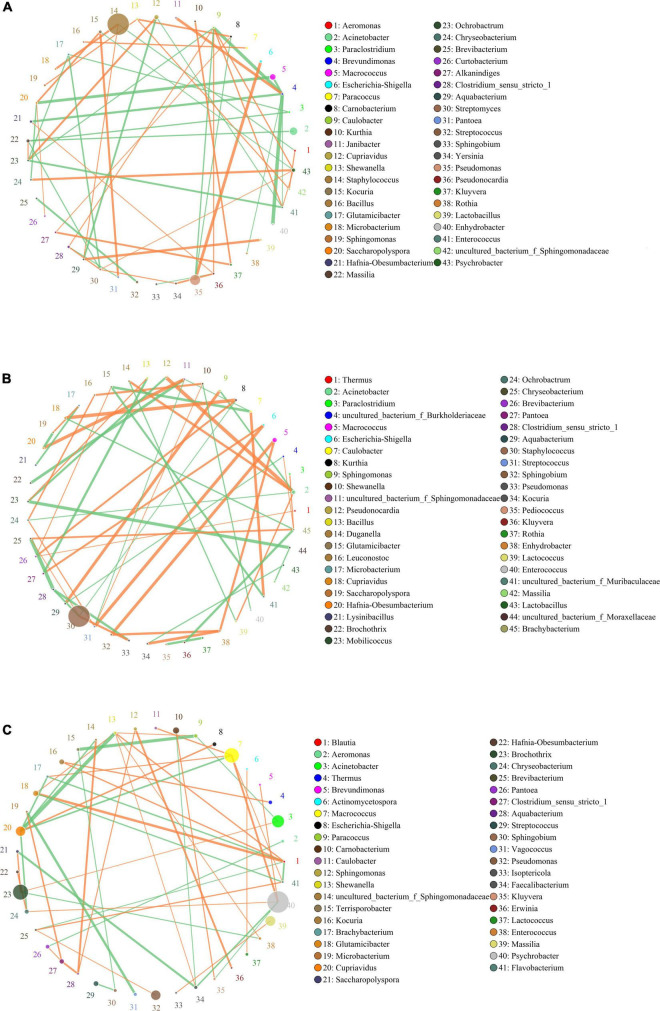
Networks of microbial interaction in the F group **(A)**, M group **(B)** and S group **(C)**. Circle is on behalf of the species, size of the circle represents the abundance. Lines represent the correlation between the two species, line thickness to represent the strength of the correlation, the color of the line: orange represents the positive correlation, green represents the negative correlation.

The networks for the bacterial community in the different groups are showed in Figure 4. Topological properties of the network in different groups are presented in Table 5. The differences in nodes, edges, density, cluster coefficient, and average path length among the three groups showed the different bacterial interactions and community structure. The network for the bacterial community in the M group had the lowest density, indicating a higher cross-talk among the resident bacteria. The cluster coefficient of the network for the bacterial community in the M group was the highest, implying that this network tended to be divided into sub-networks. The network for the bacterial community in the M group had the highest average path length value, indicating that the structure of this network was the most compact.

**TABLE 5:** Topological properties of the network in three groups.

Network	Nodes	Edges	Density	Cluster coefficient	Average path length
F group	43	54	0.0598	0.0285	3.965
M group	45	51	0.0515	0.1874	5.063
P group	41	49	0.0597	0.0512	4.217

*M, sample from the muscle tissue of bacon; F, sample from the adipose tissue of bacon; S, sample from pork skin of bacon.*

In the F group, *Brevundimonas* and *Caulobacter* were the hub genera in the bacterial community network, and had a strong negative relationship with each other. *Brevundimonas* was also negatively related to the other five genera (*Enterococcus*, *Hafnia-Obesumbacterium*, *Macrococcus*, *Enhydrobacter*, and *Rothia*), but only positively related to *Janibacter* (Figure 4A). *Staphylococcus*, the most dominant genus in the F group, had a negative relationship with *Aeromonas*, but a positive relationship with *Aquabacterium* and *Microbacterium*. No hub genera were found in the bacterial community network of the M group. *Staphylococcus*, the most dominant genus in the M group, had a negative relationship with *Clostridium_sensu_stricto*_1 and *Kurthia*, but a positive relationship with *Pseudomonas* and *Chryseobacterium* (Figure 4B). In the S group, three non-dominant genera (*Blautia*, *Shewanella*, and *Cupriavidus*) were the hub genera in the network. The most dominant genus (*Staphylococcus*) was not found in the network. *Psychrobacter*, the second most dominant genus in the S group, had a negative relationship with *Faecalibacterium* and *Kluyvera*, but a positive relationship with *Brevundimonas*, *Chryseobacterium*, and *Macrococcus* (Figure 4C).

### Correlation Between Physicochemical Characteristics and Microbial Community Composition

**FIGURE 5:**
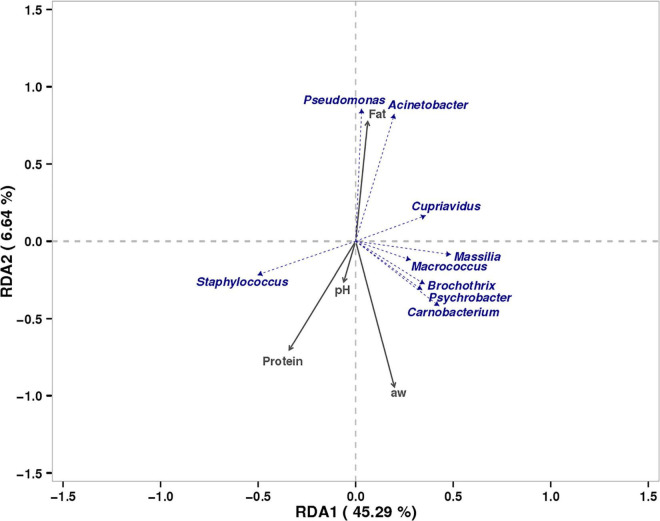
RDA of dominant bacteria and physiochemical characterization.

RDA was performed to study the relationship of physicochemical characteristics of the tissue (Supplementary Table 1) and dominant bacterial community in bacon at the genus level (Figure 5). The correlations analysis results of physicochemical characteristics of the tissue and bacon microbiota showed that RDA1 and RDA2 explained 45.29 and 6.64% of the total variance. Five dominant bacterial genera in bacon (*Staphylococcus*, *Psychrobacter*, *Macrococcus*, *Brochothrix*, *Massilia*, and *Carnobacterium*) showed positively correlation to protein content, a_*w*_, and pH, but had a negative correlation to fat content. The other two dominant bacterial genera (*Pseudomonas*and *Acinetobacter*) showed positively correlation to fat content, but had a negative correlation to protein content, a_*w*_, and pH. *Cupriavidus* showed positively correlation to protein content and pH.

## Discussion

### Bacterial Communities in Sichuan Pork Bacon Tissues

In the current study, the bacterial diversity in different parts of homemade bacon from Sichuan Province was evaluated by classical and molecular approaches. Microbiological analyses by classical approach showed that total viable count was higher in the M group. Bacterial counts showed that Staphylococcaceae and Enterobacteriaceae present in the three groups, while *Pseudomonas* and *Brochothrix* were only detected in F and S groups. These results were confirmed by molecular approaches based on PCR. The Shannon index found in this study was higher than that found for bacon from Hunan Province (Yi et al., 2016), indicating higher bacterial diversity in bacon samples in the current study. Overall, six bacterial phyla and 111 bacterial genera were detected. Among them, Firmicutes, Proteobacteria, and Actinobacteria were the first, second, and third most abundant phyla. Three phyla (Bacteroidetes, Chloroflexi, and Deinococcus-Thermus) were rare. These phyla were previously found in sausage (Wang et al., 2019), bacon from Hunan Province (Yi et al., 2016), traditional fermented pork fat (De Mandal et al., 2018), and traditional fermented fish (Zang et al., 2018). However, some other phyla (Acidobacteria, Fibrobacteres, Fusobacteria, Nitrospira, Spirochaetes, Tenericutes, and Verrucomicrobia) present in bacon from Hunan Province (Yi et al., 2016) were not detected in the current study. In total, 9 dominant genera and 102 non-dominant genera were detected in the tissues. Among them, six dominant genera (*Staphylococcus*, *Psychrobacter*, *Pseudomonas*, *Acinetobacter*, *Brochothrix*, and *Massilia*) were also detected and belonged to the top 20 genera with higher abundance in bacon from Hunan Province (Yi et al., 2016). *Sphingomonas*, the second most abundant genus in the bacon samples from Hunan Province, was present only with a relative abundance below 0.005% in the current study. Eight abundant genera (*Enhydrobacter*, *Brevibacterium*, *Methylobacterium*, *Leuconostoc*, *Chryseobacterium*, *Lactobacillus*, *Streptomyces*, and *Shewanella*) in the bacon samples from Hunan Province had relative abundances below 0.001% in the present study. Three abundant genera (*Kushneria, Parntoea*, and *Prevotelia*) in the bacon samples from Hunan Province were not present in this study. Two abundant genera (*Acinetobacter* and *Pseudomonas*) in this study were also found to be dominant in fermented pork fat (De Mandal et al., 2018).

In a previous study, Yi et al. (2016) found that the bacterial communities of bacon from three different locations significantly differed from each other, implying that geography may be a major factor in determining bacon microbial communities. Wang et al. (2018) found that the bacterial communities in Chinese dry- and smoked-cured sausages were very different from that of salami, indicating that processing technology could be another major factor in determining bacon microbial communities. Considering that bacon is naturally produced without any starter cultures, these bacteria may emerge from raw meats or processing environment. Some reports have shown that raw meat may be the origin of microbes in bacon preparation (Doulgeraki et al., 2012; Yang et al., 2017). In a previous study, *Acinetobacter* and *Pseudomonas* were also found to be dominant genera in the fresh pork samples (Yang et al., 2017). Three genera (*Aeromonas*, *Shewanella*, and *Flavobacterium*) dominated the fresh and chilled fresh pork samples (Yang et al., 2017), were present only with a relative abundance below 0.001 in our study. In the process of producing bacon, cured meat is smoked in an open environment for a long time; hence, a variety of environmental microorganisms may be involved. Then smoked bacon is usually ripened for a few months before cooking. Therefore, the microbes in bacon will be shaped continuously. Similar results were found in the production of Chinese dry- and smoked-cured sausages (Wang et al., 2018, 2019).

### Effect of Physicochemical Properties of Bacon Tissue on Bacterial Communities

The results also showed that bacteria vary greatly in different tissues. 24 significantly different taxa were detected between the tissues by a LEfSe analysis: three phyla, three classes, five orders, six families, and seven genera. The relative abundance of three phyla (Actinobacteria, Firmicutes, and Proteobacteria) significantly differentiated between the tissues (Figure 4), as also did the abundances of seven dominant genera (Figure 4). The growth of microorganisms is largely influenced by nutrition and environmental conditions (Madigan et al., 2011; Busconi et al., 2014). In the present study, bacon samples were taken from the same place and the processing environment and technology of them were generally the same. However, there are significant differences in the physicochemical characteristics of the tissues (Henry et al., 1963). Our previous study showed that physicochemical characteristics of bacon tissue greatly shaped the fungal communities in bacon (Zhang et al., 2021). Other reports showed that surface condition also affected attachment of bacteria to different tissues of meat (Greer et al., 1995; Morild et al., 2011; Zulfakar et al., 2012, 2013). In general, the growth of bacteria are greatly influenced by a variety of nutritional and environmental factors (Madigan et al., 2011). In the process of homemade traditional bacon production, raw meat serve as enrichment medium for the microorganisms. Different nutrient types of microorganisms prefer to specific nutrients and environmental conditions. Therefore, different kinds of microorganisms will grow and reproduce on the surface of different bacon tissues. In the current study, redundancy analysis showed that physicochemical characteristics of bacon tissue may be an important factor in determining bacterial communities on the surface of bacon. Because the structure and physicochemical characteristics of the various tissues on the surface of meat changes during the drying process of bacon, future studies should consider changes in bacterial populations and communities throughout the production process.

### Characteristics and Interaction of Bacteria in Meat Products

Different types of microbes, coexist in traditional foods, have different roles in food production (Zhang et al., 2018, 2021; Zhu et al., 2018; Luo et al., 2020). In the present study, we detected various kinds of bacteria in bacon, including nine dominant genera: *Staphylococcus*, *Psychrobacter*, *Pseudomonas*, *Acinetobacter*, *Macrococcus*, *Brochothrix*, *Cupriavidus*, *Massilia*, and *Carnobacterium*. *Staphylococcus*, a member of the phylum Firmicutes, currently contains more than 50 species (Fetsch and Johler, 2018). Most *Staphylococcus* spp. are known to be pathogenic and can cause severe infections (Ding et al., 2019). *Staphylococcus aureus*, the most common species, has been detected in various foods such as poultry, pork, beef, milk, and vegetables, and can cause staphylococcal food poisoning (Ding et al., 2019). Previous study showed that higher abundance of *Staphylococcus* was found in bacon samples from Yongshun and Longshan Counties in Hunan Province (Yi et al., 2016). Some *Staphylococcus* spp., such as *S. vitulinus*, *S. xylosus*, *S. carnosus*, and *S. equorum* can produce nitrosylmyoglobin (Li et al., 2013; Hu et al., 2018). Among them, *S. vitulinus* and *S. xylosus* had a stronger ability and were used in dry sausages as starter culture (Leroy et al., 2010, 2016). The *Psychrobacter* genus belongs to the phylum Proteobacteria, and is found in pork, fish, poultry, cheese, and sea ice (Gennari et al., 1992; Gonzalez et al., 2000; Pacova et al., 2001). Some species of *Psychrobacter* can produce lipase and proteases, and have been used to improve the quality of fish sauce (Zheng et al., 2017). *Psychrobacter maritimus* has been used as a novel marine probiotic for Nile tilapia fingerlings (Makled et al., 2019); however, isolated *P. immobilus* may be the cause of opportunistic infections (Gini, 1990). The Gram-positive genus *Macrococcus*, evolutionarily closely related to *Staphylococcus*, is generally regarded to be avirulent; *M. caseolyticus* has been found to be closely related to flavor formation in cheese and sausage (Mazhar et al., 2018). The genus *Carnobacterium* contains nine species, some of which can be found in the spoilage of dairy products, fish, and meat products when improperly stored; *C. maltaromaticum* has been approved for use as a preservative to inhibit *Listeria monocytogenes* in meat products (Leisner et al., 2007). Various species of genus *Cupriavidus* have been used in biotransformation (Hafuka et al., 2011), and *Cupriavidus metalliduran* was used in a multi-strain probiotic to enhance feed conversion efficiency and meat quality traits in chickens (Atela et al., 2019). Four other dominant genera (*Pseudomonas*, *Acinetobacter*, *Brochothrix*, and *Massilia*) in the current study have been found in the spoilage of fermented pork fat, bacon, and fermented fish (Yi et al., 2016; De Mandal et al., 2018). In the current study, the precise function of the bacteria is uncertain. Therefore, future studies should culture these bacteria to evaluate their impact on bacon flavor and quality formation.

## Conclusion

Numerous studies have been carried out to assess the factors affecting microbial diversity and communities in meat products. However, little is known about the effect of the tissue type on the bacterial community in pork bacon. In the current study, bacterial diversity and communities in different tissues of pork bacon from Sichuan province in China were studied using high-throughput sequencing. The results showed that physicochemical characteristics of the tissue were an important factor impacting the bacterial diversity and communities in different types of tissue. This study will deepen our understanding of the bacterial diversity of traditional pork bacon. These findings might be valuable for finding the bacterial species that can be used for improving flavor and safety of bacon in the future.

## Data Availability Statement

The original contributions presented in the study are included in the article/Supplementary Material, further inquiries can be directed to the corresponding author/s.

## Author Contributions

WZ and WG contributed conception and design of the study. YZ contributed to the experiment and manuscript revision. PW and XS performed the statistical analysis. PW and WG wrote the first draft of the manuscript. All authors contributed to the article and approved the submitted version.

## Conflict of Interest

The authors declare that the research was conducted in the absence of any commercial or financial relationships that could be construed as a potential conflict of interest.

## Publisher’s Note

All claims expressed in this article are solely those of the authors and do not necessarily represent those of their affiliated organizations, or those of the publisher, the editors and the reviewers. Any product that may be evaluated in this article, or claim that may be made by its manufacturer, is not guaranteed or endorsed by the publisher.

## Footnotes

1
https://www.r-project.org/

2
http://huttenhower.sph.harvard.edu/galaxy/

## References

[B1] Atela J. A. Mlambo V. Mnisi C. M. (2019). A multi-strain probiotic administered via drinking water enhances feed conversion efficiency and meat quality traits in indigenous chickens. *Anim. Nutr.* 5 179–184. 10.1016/j.aninu.2018.08.002 31193861 PMC6544571

[B2] Busconi M. Zacconi C. Scolari G. (2014). Bacterial ecology of PDO Coppa and Pancetta Piacentina at the end of ripening and after MAP storage of sliced product. *Int. J. Food Microbiol.* 172 13–20. 10.1016/j.ijfoodmicro.2013.11.023 24361828

[B3] De Mandal S. Singh S. S. Muthukumaran R. B. Thanzami K. Kumar V. Kumar N. S. (2018). Metagenomic analysis and the functional profiles of traditional fermented pork fat’sa-um’of Northeast India. *AMB Express.* 8:163. 10.1186/s13568-018-0695-z 30298308 PMC6175732

[B4] Ding R. X. Goh W. R. Wu R. N. Yue X. Q. Luo X. Khine W. W. T. (2019). Revisit gut microbiota and its impact on human health and disease. *J. Food Drug Anal.* 27 623–631. 10.1016/j.jfda.2018.12.012 31324279 PMC9307029

[B5] Doulgeraki A. I. Ercolini D. Villani F. Nychas G. J. E. (2012). Spoilage microbiota associated to the storage of raw meat in different conditions. *Int. J. Food Microbiol.* 157 130–141. 10.1016/j.ijfoodmicro.2012.05.020 22682877

[B6] Fetsch A. Johler S. (2018). Staphylococcus aureus as a foodborne pathogen. *Curr. Clin. Microbiol. Rep.* 5 88–96. 10.1007/s40588-018-0094-x

[B7] Gennari M. Parini M. Volpon D. Serio M. (1992). Isolation and characterization by conventional methods and genetic transformation of Psychrobacter and Acinetobacter from fresh and spoiled meat, milk and cheese. *Int. J. Food Microbiol.* 15 61–75. 10.1016/0168-1605(92)90136-Q 1622760

[B8] Gini G. A. (1990). Ocular infection caused by Psychrobacter immobilis acquired in the hospital. *J. Clin. Microbiol.* 28 400–410. 2312690 10.1128/jcm.28.2.400-401.1990 PMC269623

[B9] Gonzalez C. J. Santos J. A. Garcia-Lopez M. L. Otero A. (2000). Psychrobacters and related bacteria in freshwater fish. *J. Food Prot.* 63 315–321. 10.4315/0362-028X-63.3.315 10716558

[B10] Greer G. G. Gill C. O. Dilts B. D. (1995). Predicting the aerobic growth of Yersinia enterocolitica on pork fat and muscle tissues. *Food Microbiol.* 12 463–469. 10.1016/S0740-0020(95)80131-6

[B11] Guo X. Huang F. Zhang H. Zhang C. Hu H. Chen W. (2016). Classification of traditional Chinese pork bacon based on physicochemical properties and chemometric techniques. *Meat Sci.* 117 182–186. 10.1016/j.meatsci.2016.02.008 26994313

[B12] Hafuka A. Sakaida K. Satoh H. Takahashi M. Watanabe Y. Okabe S. (2011). Effect of feeding regimens on polyhydroxybutyrate production from food wastes by Cupriavidus necator. *Bioresour. Technol.* 102 3551–3553. 10.1016/j.biortech.2010.09.018 20870404

[B13] Henry W. E. Bratzler L. J. Luecke R. W. (1963). Physical and chemical relationships of pork carcasses. *J. Anim. Sci.* 22 613–616. 10.2527/jas1963.223613x 32704858

[B14] Hu M. Z. Yu J. S. Yu J. P. Pan Y. T. Ou Y. X. (2018). Isolation and screening of *Staphylococcus xylosus* p2 from chinese bacon: a novel starter culture in fermented meat products. *Int. J. Food Eng.* 15:20180021. 10.1515/ijfe-2018-0021

[B15] Leisner J. J. Laursen B. G. Prévost H. Drider D. Dalgaard P. (2007). Carnobacterium: positive and negative effects in the environment and in foods. *FEMS Microbiol. Rev.* 31 592–613. 10.1111/j.1574-6976.2007.00080.x 17696886 PMC2040187

[B16] Leroy S. Giammarinaro P. Chacornac J. P. Lebert I. Talon R. (2010). Biodiversity of indigenous *Staphylococci* of naturally fermented dry sausages and manufacturing environments of small-scale processing units. *Food Microbiol.* 27 294–301. 10.1016/j.fm.2009.11.005 20141949

[B17] Leroy S. Vermassen A. Talon R. (2016). “Staphylococcus: occurrence and properties,” in *Encyclopedia of Food and Health*, eds Finglas P. M. Caballero B. Toldra F. (Cambridge, MA: Academic Press), 140–145.

[B18] Li P. Kong B. Chen Q. Zheng D. Liu N. (2013). Formation and identification of nitrosylmyoglobin by *Staphylococcus xylosus* in raw meat batters: a potential solution for nitrite substitution in meat products. *Meat Sci.* 93 67–72. 10.1016/j.meatsci.2012.08.003 22926033

[B19] Li X. F. Li C. Ye H. Wang Z. Wu X. Han Y. (2019). Changes in the microbial communities in vacuum-packaged smoked bacon during storage. *Food Microbiol.* 77 26–37. 10.1016/j.fm.2018.08.007 30297053

[B20] Lozupone C. A. Hamady M. Kelley S. T. Knight R. (2007). Quantitative and qualitative diversity measures lead to different insights into factors that structure microbial communities. *Appl. Environ. Microbiol.* 73 1576–1585. 10.1128/AEM.01996-06 17220268 PMC1828774

[B21] Luo Q. Q. Zhu Y. Zhang Z. M. Cao Y. Y. Zhang W. B. (2020). Variations in fungal community and diversity in Doushen with different flavors. *Front. Microbiol.* 11:447. 10.3389/fmicb.2020.00447 32265878 PMC7099864

[B22] Madigan T. M. Martinko J. M. Stahl D. A. Clark D. P. (2011). *Brock Biology of Microorganisms*, Thirteenth Edn. San Francisco, CA: Benjamin Cummings.

[B23] Makled S. O. Hamdan A. M. El-Sayed A. M. (2019). Growth promotion and immune stimulation in Nile Tilapia, *Oreochromis niloticus*, Fingerlings following dietary administration of a novel marine probiotic, *Psychrobacter maritimus S. Probiotics* and Antimicrobial Proteins. *Probiot. Antimicrob. Proteins* 12 365–374. 10.1007/s12602-019-09575-0 31359248

[B24] Mazhar S. Hill C. McAuliffe O. (2018). The Genus *Macrococcus*: an insight into its biology, evolution, and relationship with *Staphylococcus*. *Adv. Appl. Microbiol.* 105 1–50. 10.1016/bs.aambs.2018.05.002 30342720

[B25] Morild R. K. Olsen J. E. Aabo S. (2011). Change in attachment of *Salmonella* Typhimurium, *Yersinia enterocolitica*, and *Listeria monocytogenes* to pork skin and muscle after hot water and lactic acid decontamination. *Food Microbiol.* 145 353–358. 10.1016/j.ijfoodmicro.2010.12.018 21269717

[B26] Pacova Z. Urbanova E. Durnova E. (2001). Psychrobacter immobilis isolated from foods: characteristics and identification. *Vet. Med.* 46 95–100.

[B27] Segata N. Izard J. Waldron L. Gevers D. Miropolsky L. Garrett W. S. (2011). Metagenomic biomarker discovery and explanation. *Genome Biol.* 12:R60. 10.1186/gb-2011-12-6-r60 21702898 PMC3218848

[B28] Wang H. W. Zhang X. Suo H. Y. Zhao X. Kan J. Q. (2019). Aroma and flavor characteristics of commercial Chinese traditional bacon from different geographical regions. *J. Sens. Stud.* 34:e12475. 10.1111/joss.12475

[B29] Wang X. H. Zhang Y. L. Ren H. Y. Zhan Y. (2018). Comparison of bacterial diversity profiles and microbial safety assessment of salami, Chinese dry-cured sausage and Chinese smoked-cured sausage by high-throughput sequencing. *LWT Food Sci. Technol.* 90 108–115. 10.1016/j.lwt.2017.12.011

[B30] Xiao X. Dong Y. Zhu Y. Cui H. (2013). Bacterial diversity analysis of Zhenjiang Yao meat during refrigerated and vacuum-packed storage by 454 pyrosequencing. *Curr. Microbiol.* 66 398–405. 10.1007/s00284-012-0286-1 23263225

[B31] Yang C. Che Y. Qi Y. Liang P. X. Song C. J. (2017). High-throughput sequencing of viable microbial communities in raw pork subjected to a fast cooling process. *J. Food Sci.* 82 145–153. 10.1111/1750-3841.13566 27871121

[B32] Yi L. B. Su G. R. Hu G. (2016). Diversity study of microbial community in bacon using metagenomic analysis. *J. Food Saf.* 37 1–9. 10.1111/jfs.12334

[B33] Yu A. Sun B. (2005). Flavour substances of Chinese traditional smoke-cured bacon. *Food Chem.* 89 227–233. 10.1016/j.foodchem.2004.02.029

[B34] Zang J. Xu Y. Xia W. Yu D. Gao P. Jiang Q. (2018). Dynamics and diversity of microbial community succession during fermentation of Suan yu, a Chinese traditional fermented fish, determined by high throughput sequencing. *Food Res. Int.* 111 565–573. 10.1016/j.foodres.2018.05.076 30007719

[B35] Zhang M. Qiao H. Zhang W. Zhang Z. Wen P. Zhu Y. (2021). Tissue type: a crucial factor influencing the fungal diversity and communities in Sichuan pork bacon. *Front. Microbiol.* 12:655500. 10.3389/fmicb.2021.655500 34248870 PMC8268000

[B36] Zhang W. B. Luo Q. Q. Zhu Y. Ma J. Cao L. Yang M. (2018). Microbial diversity in two traditional bacterial douchi from Gansu province in northwest China using Illumina sequencing. *PLoS One* 13:e0197527. 10.1371/journal.pone.0194876 29570735 PMC5865742

[B37] Zheng B. Liu Y. He X. Hu S. Li S. Chen M. (2017). Quality improvement on half-fin anchovy (Setipinna taty) fish sauce by Psychrobacter sp. SP-1 fermentation. *J. Sci. Food Agric.* 97 4484–4493. 10.1002/jsfa.8313 28294344

[B38] Zhu Y. Cao Y. Y. Yang M. Wen P. C. Cao L. Ma J. (2018). Bacterial diversity and community in Qula from the Qinghai-Tibetan Plateau in China. *PeerJ* 6:e6044. 10.7717/peerj.6044 30568858 PMC6286660

[B39] Zulfakar S. S. White J. D. Ross T. Tamplin M. L. (2012). Bacterial attachment to immobilized extracellular matrix proteins *in vitro*. *Int. J. Food Microbiol.* 157 210–217. 10.1016/j.ijfoodmicro.2012.05.007 22647675

[B40] Zulfakar S. S. White J. D. Ross T. Tamplin M. L. (2013). Effect of pH, salt and chemical rinses on bacterial attachment to extracellular matrix proteins. *Food Microbiol.* 34 369–375. 10.1016/j.fm.2013.01.010 23541204

