# Supplementary material for: Influence of Tissue Type on the Bacterial Diversity and Community in Pork Bacon
Source: Front Microbiol. 2021 Dec 3;12:799332. doi: 10.3389/fmicb.2021.799332 (PMC8678503; doi:10.3389/fmicb.2021.799332)
Supplement: Supplementary file 1 [file Table_1.docx]

Table S1 Physiochemical characterization of bacon

| Sample ID | Protein content  (g/100 g) | Fat content  (g/100 g) | Moisture content  (g/100 g) | Salt content  (g/100 g) | Aw | pH |  |
| --- | --- | --- | --- | --- | --- | --- | --- |
| M1 | 53.75±2.29 ^ab^ | 5.45±0.15 ^a^ | 34.89±1.24 ^a^ | 5.21±0.25 ^a^ | 0.832±0.021 ^ab^ | 5.6±0.1 ^ab^ |  |
| M2 | 54.32±1.46 ^a^ | 5.56±0.18 ^a^ | 33.38±1.35 ^a^ | 5.35±0.21 ^a^ | 0.851±0.032 ^a^ | 5.7±0.1 ^a^ |  |
| M3 | 55.48±2.31 ^a^ | 5.39±0.15 ^a^ | 35.19±2.48 ^a^ | 5.38±0.18 ^a^ | 0.873±0.042 ^a^ | 5.9±0.3 ^a^ |  |
| M4 | 55.48±3.26 ^a^ | 5.71±0.21^a^ | 34.53±2.46 ^a^ | 5.45±0.31 ^a^ | 0.824±0.031 ^ab^ | 5.8±0.2 ^a^ |  |
| M5 | 55.19±2.59 ^a^ | 5.39±0.13^a^ | 34.43±1.52 ^a^ | 5.41±0.14 ^a^ | 0.853±0.032 ^a^ | 5.9±0.3 ^a^ |  |
| M6 | 53.49±2.48^a^ | 5.78±0.26^a^ | 35.13±2.29 ^a^ | 5.38±0.19 ^a^ | 0.821±0.023 ^ab^ | 5.7±0.1 ^a^ |  |
| M_mean_ | 54.61±1.89 ^A^ | 5.54±0.23 ^C^ | 34.59±2.56 ^A^ | 5.36±0.19 ^A^ | 0.843±0.022 ^A^ | 5.8±0.1 ^A^ |  |
| F1 | 5.12±0.13 ^ab^ | 83.25±1.31 ^ab^ | 8.12±0.21 ^a^ | 2.15±0.09 ^a^ | 0.761±0.022 ^a^ | 5.3±0.5 ^ab^ |  |
| F2 | 5.35±0.31 ^a^ | 84.15±2.13 ^a^ | 8.51±0.33 ^a^ | 2.31±0.12 ^a^ | 0.791±0.032 ^a^ | 5.8±0.2 ^a^ |  |
| F3 | 5.09±0.21 ^ab^ | 84.31±2.18 ^a^ | 8.09±0.21 ^a^ | 2.43±0.11 ^a^ | 0.752±0.043 ^a^ | 5.9±0.2 ^a^ |  |
| F4 | 5.41±0.12 ^a^ | 85.34±1.29 ^a^ | 8.31±0.43 ^a^ | 2.26±0.23 ^a^ | 0.793±0.012 ^a^ | 5.7±0.3 ^a^ |  |
| F5 | 5.21±0.22 ^ab^ | 84.19±2.32 ^a^ | 8.15±0.13 ^a^ | 2.18±0.21 ^a^ | 0.772±0.021 ^a^ | 5.7±0.4 ^a^ |  |
| F6 | 5.34±0.12 ^a^ | 83.14±1.95 ^ab^ | 8.22±0.11 ^a^ | 2.51±0.45 ^a^ | 0.783±0.032 ^a^ | 5.6±0.4 ^a^ |  |
| F_mean_ | 5.25±0.13 ^C^ | 84.06±1.80 ^A^ | 8.22±0.16 ^B^ | 2.31±0.14 ^B^ | 0.774±0.023 ^B^ | 5.7±0.2 ^A^ |  |
| S1 | 29.21±1.51 ^a^ | 24.56±2.32 ^a^ | 36.51±1.63 ^a^ | 5.13±0.21 ^a^ | 0.852±0.054 ^a^ | 5.7±0.3 ^a^ |  |
| S2 | 29.81±2.35 ^a^ | 23.91±3.65 ^a^ | 36.18±1.45 ^a^ | 5.32±0.13 ^a^ | 0.862±0.042 ^a^ | 5.3±0.4 ^ab^ |  |
| S3 | 28.75±1.26 ^a^ | 24.28±1.31 ^a^ | 36.36±1.57 ^a^ | 5.52±0.31 ^a^ | 0.891±0.071 ^a^ | 5.9±0.3 ^a^ |  |
| S4 | 29.36±2.45 ^a^ | 24.59±2.23 ^a^ | 35.81±3.43 ^a^ | 5.61±0.26 ^a^ | 0.804±0.031 ^ab^ | 5.8±0.2 ^a^ |  |
| S5 | 29.69±3.24 ^a^ | 24.75±1.92 ^a^ | 37.32±1.35 ^a^ | 5.45±0.31 ^a^ | 0.831±0.072 ^a^ | 5.7±0.5 ^a^ |  |
| S6 | 28.90±2.51 ^a^ | 25.35±1.71 ^a^ | 36.81±2.23 ^a^ | 5.38±0.43 ^a^ | 0.853±0.052 ^a^ | 5.8±0.4 ^a^ | |
| S_mean_ | 29.29±1.42^B^ | 24.57±1.48 ^B^ | 36.49±1.52 ^A^ | 5.41±0.26 ^A^ | 0.842±0.041 ^A^ | 5.7±0.2 ^A^ | |

Note: M1-M6, sample from the muscle tissue of bacon; F1-F6, sample from the adipose tissue of bacon; S1-S6, sample from pork skin of bacon.

Lowercase letters indicate Duncan’s pairwise differences between samples from different producers (p<0.05);Uppercase letters indicate Duncan’s

pairwise differences between samples from different tissue of bacon (p<0.05).
